# A six-year grazing exclusion changed plant species diversity of a *Stipa breviflora* desert steppe community, northern China

**DOI:** 10.7717/peerj.4359

**Published:** 2018-02-13

**Authors:** Xing Wang, Xinguo Yang, Lei Wang, Lin Chen, Naiping Song, Junlong Gu, Yi Xue

**Affiliations:** 1 Key Laboratory for Restoration and Reconstruction of Degraded Ecosystems in Northwestern China of the Ministry of Education, United Center for Ecology Research and Bioresource Exploitation in Western China, Ningxia University, Yinchuan, China; 2 School of Agriculture Ningxia University, Yinchuan, China

**Keywords:** Species-area curve, Spatial heterogeneity, Diversity patterns, Variance partitioning

## Abstract

Excluding grazers is one of most efficient ways to restore degraded grasslands in desert-steppe communities, but may negatively affect the recovery of plant species diversity. However, diversity differences between grazed and fenced grasslands in desert-steppe are poorly known. In a *Stipa breviflora* desert steppe community in Northern China, we established six plots to examine spatial patterns of plant species diversity under grazed and fenced conditions, respectively. We addressed three aspects of species diversity: (1) The logistic, exponential and power models were used to describe the species-area curve (SAR). Species richness, abundance and Shannon diversity values change differently with increasing sampling areas inside and outside of the fence. The best fitted model for SAR was the logistic model. Excluding grazers had a significant impact on the shape of SAR. (2) Variograms was applied to examine the spatial characteristics of plant species diversity. We found strong spatial autocorrelations in the diversity variables both inside and outside the fence. After grazing exclusion, the spatial heterogeneity decreased in species richness, increased in abundance and did not change in Shannon diversity. (3) We used variance partitioning to determine the relative contributions of spatial and environmental factors to plant species diversity patterns. Environmental factors explained the largest proportion of variation in species diversity, while spatial factors contributed little. Our results suggest that grazing enclosures decreased species diversity patterns and the spatial pattern of the *S. breviflora* desert steppe community was predictable.

## Introduction

Human disturbance and global climate change pose severe threats to biodiversity and ecosystem functioning of grasslands (Bai et al., 2012; Shao et al., 2016). The dramatic degradation of grasslands has prompted efforts to restore them through ecological methods such as planting shrubs, excluding grazers, introducing species and changing land use practices. The ultimate goal of restoration should be to maintain biological diversity and the associated ecological services (Peco et al., 2012; Wu et al., 2014). A simple and common restoration method to stimulate self-recovery is by excluding grazers. However, grazing enclosures can have negative effects on plant species diversity due to litter accumulation, altered competition for water and light, and competitive exclusion of species (Guo, 2005; Wu & Thirgood, 2009; Jing, Cheng & Chen, 2013). The change in species diversity patterns are the outcome of multiple mechanisms: such as environment filtering, biotic interactions and/or dispersal limitation (Crist & Veech, 2006; Chiarucci et al., 2010; Hillerislambers et al., 2012). Understanding which mechanisms underlie the change in species diversity due to grazing exclusion is critical to an informed management of restoration efforts in steppe-grasslands.

Research into the effect of grazing on species diversity pattern has focused on disturbance characteristics, water and nutrient availability, and topography of arid and semi-arid grasslands (Reitalu et al., 2012; Lan et al., 2015; Li et al., 2015). In grasslands, diet selection by grazing animals is a critical factor shaping diversity patterns. For example, a U-shaped diversity-grazing intensity relationship was detected because the palatable species (mainly forbs) are the major contributors to species diversity and those species were most severely damaged at intermediate grazing intensity (Wan et al., 2015). Heavy grazing disturbance altered species composition, usually favouring annual plants which were unpalatable species (Rutherford & Powrie, 2013). Furthermore, the combination of grazing stress and abiotic stress can have complex impacts on plant species diversity. For example, the combination of moderate grazing and periodic drought can enhance plant species diversity and fine-scale spatial heterogeneity in a savanna understory (Porensky & Young, 2013). Grazing and water availability were key factors of diversity patterns in Mediterranean grassland communities (Rota et al., 2016).

Moreover, grazing effects also interact with topography and soil appears to impact abundance of dominant species, and these dominant species had a significant effect on diversity (Fırıncıoğlu et al., 2009). Species richness increased with soil PH, moisture and bulk density inside fences, but grazing decoupled the relationships between species diversity and soil properties in semiarid grasslands (Jing et al., 2017). Grassland restoration in arid region that allows community self-organization may have more complex outcomes in response to fencing due to nonlinear trajectories and interactions between species (Lawley et al., 2013). Internal dynamics may play a more significant role in determining species diversity at local scales than external factors after a long-term grazing enclosure (Lawley et al., 2013). In sum, two essential processes have been identified as being important in determining species diversity patterns: the characteristics of grazing disturbance and the recovery and colonizing processes of the disturbed organisms (Wan et al., 2015). Any factors that impact these two processes can potentially affect the diversity patterns.

Additionally, species richness from a particular region is related to numerous factors and the effect of management type or different ecological factors on species richness is scale-dependent (FernándezLugo et al., 2011; Li et al., 2015; Mardari, 2016). Species–area relationship (SAR) is among the best-documented pattern in community ecology and is frequently used to study diversity patterns across spatial scales (FernándezLugo et al., 2011; Wilson, Peet & Dengler, 2012; Li et al., 2015). Moreover SAR can be used to understand the relationships between diversity and the above mentioned ecological factors, using the power-law function *S* = *cA*^*z*^ (*S* = species richness, *A* = area, *c*, intercept; *z*, slope), where *c* and *z* are constants, the parameters *c* and *z* can be regarded as a measure of changing in species richness across multiple scales (Sandel & Corbin, 2012). Species–area curves are useful to analyze disturbances as grazing on species richness, allowing us to partition of spatial components of species diversity (Loreau, 2000). Because grazing may have a pronounced effect on species abundance distributions, community productivity and composition in comparison to grazing exclusion (Zhang et al., 2011; Dong et al., 2015; Ren et al., 2015), these factors mentioned above can alter the parameters of SAR (Chiarucci et al., 2006; Dolnik & Breuer, 2008). However, results into grazing effect on SAR are not consistent (Lande, Devries & Walla, 2000; FernándezLugo et al., 2011; Li et al., 2015), and it is necessary to study this aspect of species diversity in different locations and situations.

The studies reviewed above have led ecologists to better understand plant species diversity between grazed and fenced grasslands. Previous studies in desert steppe communities have examined species–area relationships and the scale dependence of species diversity patterns (He et al., 2006; Jiang et al., 2013). However, it is unknown how plant species diversity is distributed spatially within grazed and fenced desert steppe communities, respectively, and to what extent the species diversity patterns are explained by environmental and spatial factors? In this study, we aimed to examine spatial patterns of species diversity between grazed and fenced areas (within grazing exclosures) in a desert steppe community. We established the grazed and fenced treatments in one *S. breviflora* steppe (typical natural steppe) site in northern China. Because *S. breviflora.* is a species typical to continental steppes, a mature ecosystem dominated by this species can be considered the desired state post-restoration (Bai et al., 2012; Jing, Cheng & Chen, 2013). To assess how grazing exclosure affects spatial patterns in plant diversity, we asked the following questions: (1) Does grazing exclosure alter species–area relationships compared to grazed areas? (2) What are the spatial patterns in species diversity in grazed and fenced grasslands? (3) To what extent do spatial (modeled from the spatial coordinate data) and environmental factors contributed to the observed species diversity patterns?

## Materials and Methods

### Study area

The experiment was conducted in Maerzhuang village (37°27′30″–37°37′30″N and 106°37′30″–106°56′15″E, and altitude 1,450 m, within Yanchi country, Ningxia Hui Autonomous Region, northern China. The area is located in the southwestern fringe of Mu US Sandy Land and in the mid-western regions of the Loess Plateau. Harsh climatic conditions in this area cause land desertification, which is exacerbated by overgrazing. The mean annual temperature is 8.1 °C, and temperature ranges from −8.7 °C in January to 22.4 °C in July. The annual precipitation is 295.1 mm, with rainfall occurring mainly between June and September (Tang, An & Shangguan, 2015). The annual average evaporation capacity is 2,136 mm. The main soil types in this area are sierozem, loess, and orthi-sandic entisols, all of which have low fertility and loose structure (Liu et al., 2014). The representative vegetation includes *S. breviflora*, *Leymus chinensis*, and *Agropyron cristatum* communities, but the vegetation has been greatly altered by long-term overgrazing (Xie et al., 2015).

### Experimental design and data collection

**Figure 1:**
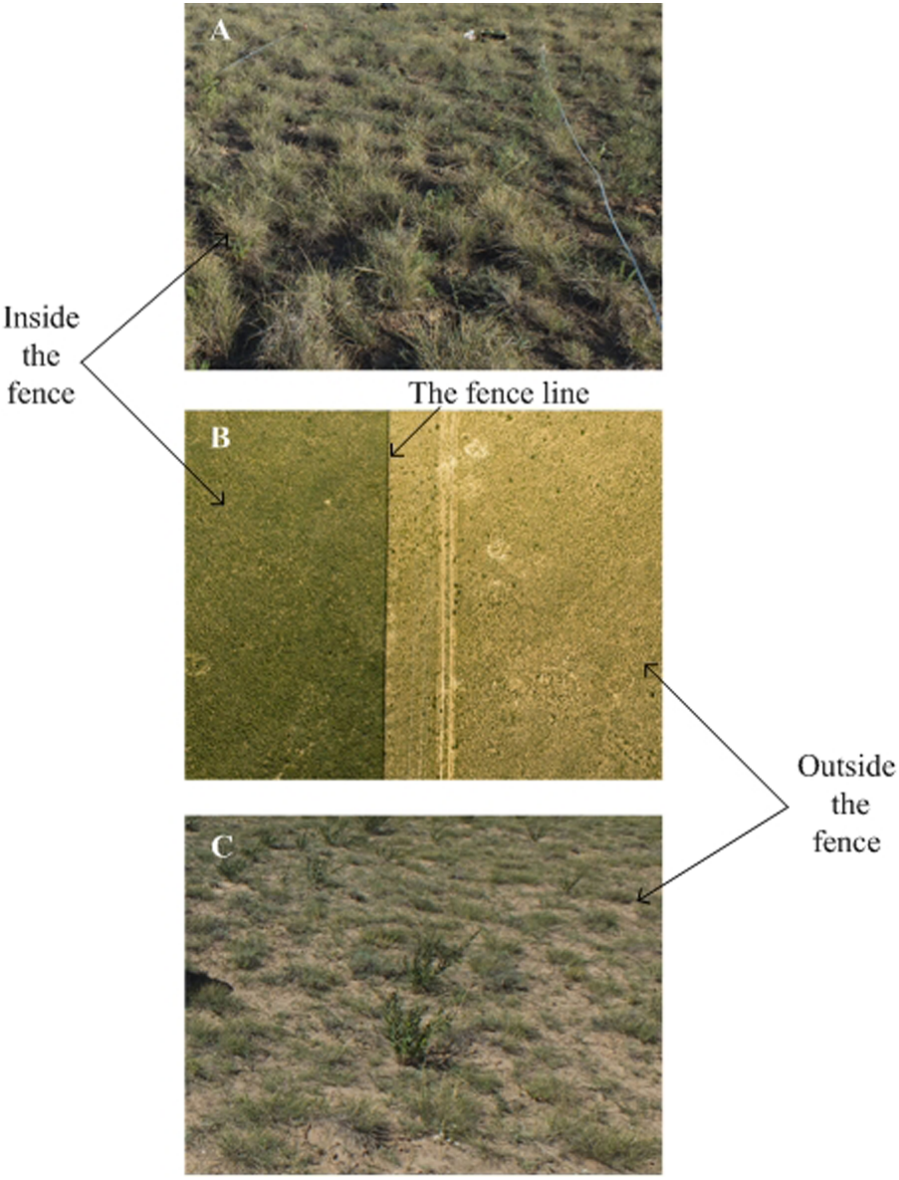
The landscape of study sites (The middle picture was taken using Unmanned aerial vehicle (DJIS1000 + Canon 5D Mark III)). The fence line is clearly visible with a sharp contrast between vegetation density and cover inside and outside. Photos by Lei Wang. (A) Inside the fence; (B) the whole study site; (C) outside the fence.

In late July 2016, we selected one site with two treatments, grazed vs. fenced. Inside the fenced grassland, livestock had been abandoned for six years (from 2010 to 2016) and outside the fenced grassland grazing was allowed all the year around at a level of grazing intensity (1.5 sheep ha-1) (Fig. 1). The predominant plant species at the site were *S. breviflora*. (perennial grass), *Lespedeza potaninii Vass*, *Convolvulus ammannii Desr* and *Cleistogenes squarrosa* (Trin.). Within the grazed and fenced conditions, we established three 50 × 50 m plots, 500 m apart from each other. We established a total of six plots (two treatments with three plots each).

### Plant species investigation and soil sampling

Three 5 × 5 m subplots were established in grazing and fenced treatments. We conducted vegetation surveys within each subplot, which were further divided into 100 (50 cm × 50 cm) units for sampling. In each sampling unit, we recorded the number of species, plant height, cover, and abundance for each plant species. The specific operations for a detailed vegetation survey and biomass measurement were given by a study in the same region (Waldén & Lindborg, 2016). During the study period, time domain reflectometry (TDR) and a stratameter were used to detect soil water content and soil compaction at the depth 0–10 cm, which we used as soil environmental factors in subsequent analyses. We recorded soil compaction because trampling from domestic animals can compact soil and alter soil structure, soil bulk density, and the transport of oxygen and water, which may be detrimental to plants in grassland (Klink et al., 2015). Then, we collected one random soil samples in each sampling unit with a 5-cm diameter soil auger from 0–10 cm depth. We collected a total of 100 soil samples and established 100 vegetation sampling units within each 5 m × 5 m subplot. We chose this size for our subplots because preliminary sampling for soil and vegetation, using nested sampling design, showed that the 5 m^2^ is a saturation point of species richness, and the minimum size of sampling unit should be 0.5 m^2^ (Fig. S1). Moreover, competitive exclusion among co-occurring species can occur at the finest spatial scale, where individuals of different species are more competitive with neighbors for resources and space (Luzuriaga et al., 2012). For example, coexisting species at 50 cm × 50 cm showed a convergence pattern caused by environmental filtering in dry semi-natural grasslands (Bello et al., 2013).

## Data Analysis

We used species richness, species abundance, and the Shannon’s index to quantify diversity. Species richness was measured using the number of species in each sampling unit (Myers & Bazely, 2003). Abundance for each species is defined as the number of all individuals (Myers & Bazely, 2003). Shannon diversity as a synthetic measure of community structure was calculated using the Vegan package (ver. 2.0–2) in R (Oksanen, 2016). All statistical analyses were performed using the mean of different variables for the three subplots in both grazed and fence grassland.

Different methods were applied to address the questions mentioned in Introduction. For questions 1, species richness, abundance and Shannon diversity index were calculated in square sampling units that ranged from 0.5 m × 0.5 m to the whole 5 m × 5 m subplot. The expected species–area curve (null model) was derived under the hypothesis that all species in this study are randomly distributed (He & Legendre, 2002). The logistic, exponential and power models were used to describe the species–area curve (SAR). For the species–area curve (SAR), the statistical criterion of the best fitted model is the sum of squares of the residuals (Wang et al., 2008). In addition, in order to test whether models were significantly different, the 95% confidence intervals of the different model parameters were given (Wang et al., 2008).

For question 2, we used a spatial variogram to examine how spatial distance influenced the distribution of plant species diversity. Variograms are common method to detect spatial heterogeneity of plant species diversity (He et al., 2006; Wang et al., 2008). A variogram can be described as the relationship between semi-variance and distance lags (Xie et al., 2015). The standard formula for the semi-variance (*γ*(*h*)) is: (Eq. (1))

(1) \begin{eqnarray*}\gamma (h)= \frac{1}{2N(h)} \sum _{i=1}^{N(h)}[Z({x}_{i})-Z({X}_{i}+h)]^{2}\end{eqnarray*}

where *N* (*h*) is the total amount of sample pairs separated by distance *h*; *Z*(*x*_*i*_ + *h*) and *Z*(*x*_*i*_) are the measured values at the location *x*_*i*_ + *h* and *x*_*i*_, respectively. Three basic parameters in variograms were used to interpret the spatial characteristics of a variable. (1) The range (*A*) is the spatial scale at which the spatial influence disappears; (2) the nugget variance (*C*_0_) is either the spatial variation or the random error occurring at a finer scale than the sampling interval; and (3) the sill (*C* + *C*_0_) is the semi-variance value that the variograms reaches at the range. This value is the total variance of a variable, including structural variance (*C*) and nugget variance (*C*_0_).

For question 3, we used variation partitioning analysis to test the relative importance of spatial and environmental variables in explaining plant species diversity in the *S. breviflora* desert steppe community (Wang et al., 2008). Variation partitioning examines the partitioning of variation in species diversity based on Hellinger-transformed species data and standardization-transformed environmental data (Bello et al., 2013; Wang et al., 2013). The spatial eigenvectors (as a proxy for spatial factors) were obtained through Principal Coordinates of Neighbor Matrices (PCNM) analysis using the geographical coordinates of all samples (Lewis, Pakeman & Marrs, 2014). For a complete description of the method, see Dray, Legendre & Peres-Neto (2006). Environmental variables included soil characteristics (organic carbon, water content and particle size) and aboveground biotic factors (cover and density of *S. breviflora*). Since there was strong collinearity among some environment variables, we first removed environmental variables that were highly correlated with other variables and then used a forward selection (“forward.sel”) function in the R package packfor 0.0–8 to retain environment variables with a significant effect on plant diversity (Wang et al., 2016). Similarly, forward selection was also performed for the PCNM variables. The significant environmental and PCNM variables retained (*α* = 0.05, Monte Carlo permutation, *n* = 999) in the final model were then applied to partition variation in plant diversity into four fractions: [a] = variation explained by the environmental factors independent of space, [b] = variation explained by the spatially structured environment factors, [c] = variation explained by spatial factors independent of any environment factors, [d] = residual variation (Simpson et al., 2016).

## Results

### Species diversity inside and outside the fence

For both inside and outside the fence, the best fitted model was the logistic model, with the lowest residual sum of squares (Table 1). Grazing exclusion had a significant effect on the parameters of the SAR models. For instance, there was no overlap in the 95% confidence intervals for observed *α* and *β* parameters in the logistic models inside and outside of the fence (Table 1).

**Table 1:** Comparison of three species (S)–area (A) models (SAR) between outside (Out) and inside (In) of the fence: power, exponential and logistic. ‘sum(residuals)’ is the sum of squared residuals after fitting the given model, and ‘conf.interval’ provides the 95% confidence intervals of the parameter values. The logistic model is the best one to fit species–area curves, whereas the exponential model is the worst.

	Power model		Exponential model		Logistic model	
	*S* = *α* + *A*^*β*^(Out)∕*S* = *αA*^*β*^ (In)		*S* = *α*ln(*A*) + *β*		*S* = *β*∕(1 + exp((*δ* − log(*A*))∕*α*))	
	Parameters ± conf.interval	sum(residuals)	Parameters ± conf.interval	sum(residuals)	Parameters ± conf.interval	sum(residuals)
^a^Out true SAR	*α* = 17.03 ± 0.20	67.4	*α* = 0.05 ± 0.004	135.17	*α* = 1.99 ± 0.021	0.407
	*β* = 0.45 ± 0.01		*β* = 19.68 ± 0.24		*β* = 26.58 ± 0.07	
					*δ* = 0.115 ± 0.01	
^b^Out expected	*α* = 21.14 ± 0.32	127	*α* = 0.01 ± 0.004	149.63	*α* = 0.51 ± 0.008	1.355
SAR	*β* = 0.25 ± 0.03		*β* = 23.14 ± 0.25		*β* = 24.04 ± 0.012	
					*δ* = − 0.02 ± 0.009	
^a^In true SAR	*α* = 13.77 ± 0.107	10.9	*α* = 0.062 ± 0.004	113.5	*α* = 2.601 ± 0.0473	0.729
	*β* = 0.114 ± 0.002		*β* = 17.80 ± 0.22		*β* = 28.24 ± 0.220	
					*δ* = 0.115 ± 0.01	
^b^In expected	*α* = 19.91 ± 0.413	117	*α* = 0.014 ± 0.004	161.1	*α* = 0.53 ± 0.009	1.573
SAR	*β* = 0.04 ± 0.005		*β* = 22.07 ± 0.26		*β* = 23.05 ± 0.014	
					*δ* = 0.05 ± 0.01	

**Notes.**

a indicates the true species–area curve.

b indicates the expected species–area curve.

Moreover, the expected SAR curves were significantly different from the observed SAR curves at the same conditions (inside or outside) (Fig. S2, Table 1). We found consistent differences in abundance-area and density-area curves, with curves inside the fence having higher values at all sampling scales (Fig. S3). The relation between area and Shanon-diversity varied little inside and outside the fence for all sampling scales (Fig. S4, Table 2). The Shanon diversity-area curves were well fitted by all three models both inside and outside the fence.

**Table 2:** Comparison of three Shannon diversity (S)-area (A) models: power, exponential and parabolic between outside (Out) and inside (In) the fence. ‘sum(residuals)’ is the sum of squared residuals after fitting the given model, and ‘conf.interval’ indicates the 95% confidence intervals of the paremeter values. The exponential model is the best fit to diversity-area curve for Shannon’s index outside the fence, whereas the power model provided the best fit for Shannon’s diversity index inside the fence.

	Power model		Exponential model		Parabolic model	
	*S* = *α* + *A*^*β*^(Out)∕*S* = *αA*^*β*^(In)		*S* = *α*ln(*A*) + *β*		*S* = *α*ln(*A*)^2^ + *β*ln(*x*) + *δ*	
	Parameters ± conf.interval	sum(residuals)	Parameters ± conf.interval	sum(residuals)	Parameters ± conf.interval	sum(residuals)
Out	*α* = 1.18 ± 0.009	0	*α* = 0.0025 ± 0.00	0	*α* = − 6.1 × 10^−6^ ± 0.00	0.1
	*β* = 0.063 ± 0.002		*β* = 2.31638 ± 0.005		*β* = 0.0031 ± 0.0003	
					*δ* = 2.306 ± 0.007	
In	*α* = 1.764 ± 0.0667	4	*α* = 0.0044 ± 0.001	5	*α* = − 1.01 × 10^−4^ ± 0.00	4.4
	*β* = 0.0801 ± 0.01		*β* = 2.1459 ± 0.045		*β* = 0.0146 ± 0.003	
					*δ* = 1.97 ± 0.065	

### Spatial structure of species diversity

**Figure 2:**
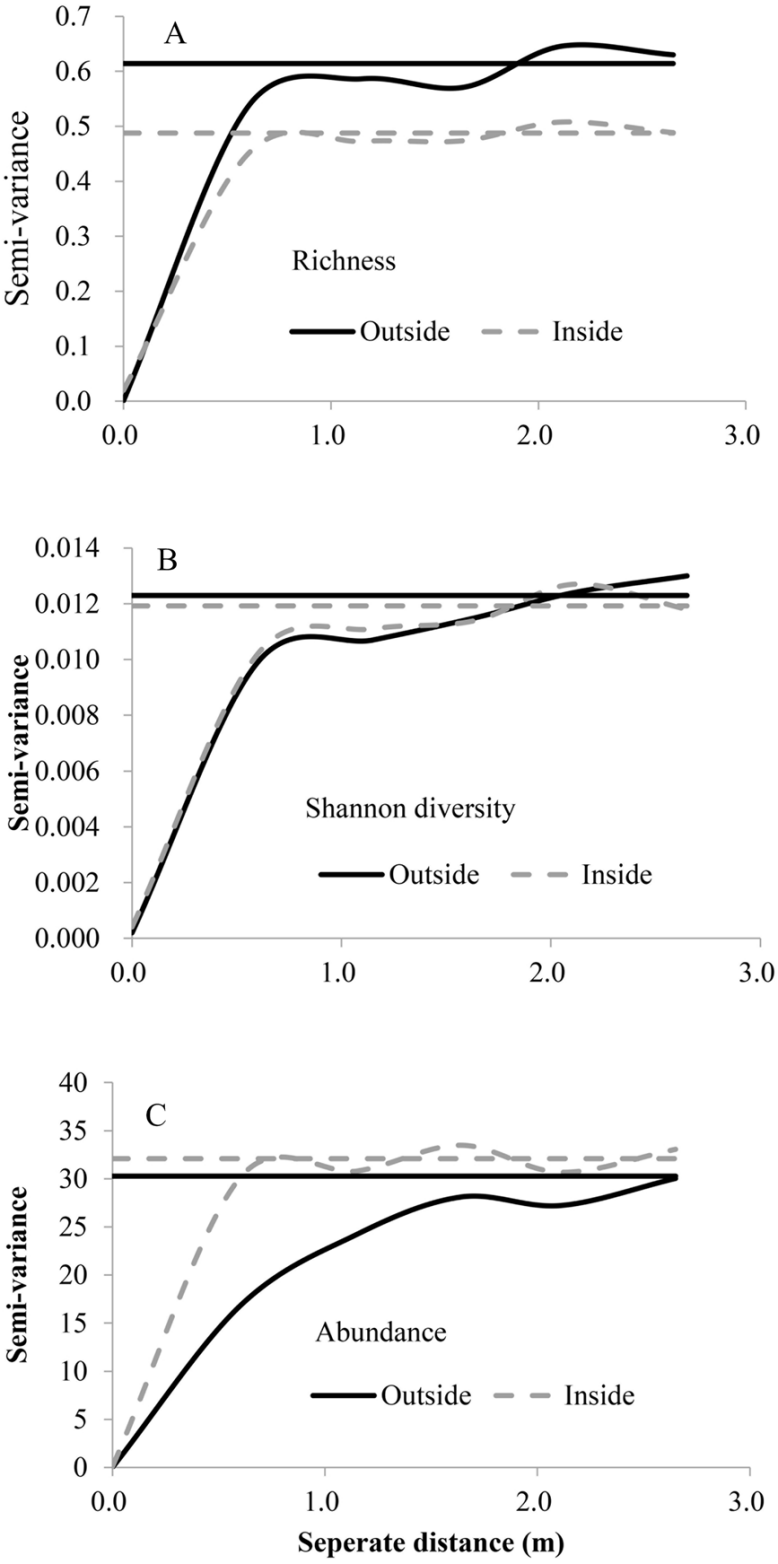
Variograms of species richness, Shannon diversity and abundance outside and inside the fence. The horizontal line indicates the overall variance of the variables. (A) Spatial heterogeneity of plant species richness; (B) spatial heterogeneity of plant Shannon diversity; (C) spatial heterogeneity of plant species abundance.

For both inside and outside of the fence, the exponential model was the best fit for three diversity indices (Fig. 2, Table 3), the range (*A*_0_) showed that fine scale effects (around 1 m) dominate, the values of *C*∕*C*_0_ + *C* were relatively large and showed relatively strong spatial autocorrelation, and the nugget variance was low. Abundance had higher spatial heterogeneity than richness and Shanon diversity.

**Table 3:** Analysis of the spatial structure for species diversity, abundance and Shannon diversity between inside (In) and outside (Out) the fence.

Variable	Exclusion	Model	*C*_0_	*C*_0_ + *C*	*C*∕*C*_0_ + *C*	*A*_0_(m)	*R*^2^
Species richness	Out	Exponential	0.001	0.614	0.98	0.30	0.604
	In	Exponential	0.022	0.488	0.95	0.25	0.617
Abundance	Out	Exponential	0.010	30.29	1.00	0.73	0.970
	In	Exponential	0.100	32.09	0.997	0.22	0.340
Shannon diversity	Out	Exponential	0.000	0.012	0.984	0.42	0.704
	In	Exponential	0.000	0.012	0.966	0.33	0.692

Spatial heterogeneity was affected after grazing exclusion (decline in richness, increase in abundance and no change in Shanon diversity), while the intensity of spatial dependence was not (Fig. 2). All analyzed diversity variables indicated that the spatial structure was different after grazing exclusion.

### Spatial patterns of species diversity

The total variation ([*a* + *b* + *c*]) of three diversity variables data (richness, abundance and Shannon diversity) explained by environmental and spatial structure factors outside was greater than that inside the fence (Table 4). Inside the fence, the variation explained by the environmental factors (pure effect) [a] increased, while the variation explained by the spatially structured environment factors [b] decreased in comparison to that outside the fence. The pure effect of environment variables [a] was statistically significant for three diversity variables and the variation explained by spatial factors independent of any environment factors [c] was only statistically significant for abundance outside of the fence. The environmental variables were significantly related to plant species diversity both inside and outside the fence (Table S3). For example, the silt content explained 79.0% of total variation in species richness outside the fence, whereas coarse sand contributed 59% of total variation in species richness inside of the fence (Tables S3, S4).

**Table 4:** Relative percentage of variation partitioning of species richness, Shannon diversity and abundance outside (Out) and inside (In) the fence.

Variation partitioning	Shannon diversity		Species richness		Abundance	
	Out	In	Out	In	Out	In
[a] pure environment	43.93%^**^	57.91%^**^	35.40%^**^	46.52%^**^	33.29%^**^	43.94%^**^
[b] space + environment	49.82%	23.53%	47.20%	21.62%	36.79%	8.89%
[c] pure space	0.00%	0.69%	1.20%	1.72%	4.21%^*^	2.39%
[d] undetermined	6.27%	17.86%	16.20%	30.14%	25.70%	44.79%

**Notes.**

* represent *P* < 0:001.

** represent *P* < 0.005.

## Discussion

### Species diversity–area relationship

When a plant community is in equilibrium, its species–area relationship should be well fitted by a power function (Wang et al., 2008). However, the logistic model was the best to describe the SAR curves both inside and outside of the fence (Table 1). This suggests that the species composition in this study was not in a state of equilibrium. The expected SAR curves were significantly different from the observed SAR curves both inside and outside of the fence (Fig. S2, Table 1), showing that species in this typical desert steppe ecosystems are not randomly distributed.

Species area curves present a “genuine law” of species diversity (He & Legendre, 2002; Panitsa, Tzanoudakis & Sfenthourakis, 2008). Previous studies have reported that species–area relationships show small but significant differences between fenced and grazed areas, and this difference is more apparent at smaller spatial scales in grazed conditions (FernándezLugo et al., 2011). In our study, grazing exclusion had a significant impact on the diversity -area curves at fine scales (Fig. S2). For the grazed grasslands, the abundance of more palatable species may decline, and unpalatable species may increase due to direct herbivory. Furthermore, spatial distributions of species could change because of positive interactions, termed ‘associational resistance’, at the neighbourhood scale in response to disturbance (e.g., perennial and annual plants). For the fenced grassland, self-organization defined as species’ internal dynamics and interactions was critical in determining diversity patterns (Ren et al., 2015). Therefore, spatial distribution of species and the abundance are two direct factors that immediately determine species–area relationships, while other factors (environmental factor and biotic factors) can indirectly affect the spatial distributions of species and abundances (He & Legendre, 2002).

### The spatial structure of species diversity

Spatial heterogeneity is an important property of natural ecosystems (Ren et al., 2015). In our study site, the relatively higher structural variance to sill value (*C*∕(*C* + *C*0)) for diversity indicated that the spatial heterogeneity mainly came from spatial autocorrelation (He et al., 2006). This pattern held true for all sampling scales in our study (Fig. 2, Table 3). Fine-scale spatial heterogeneity is considered a conspicuous characteristic of grasslands and this spatial heterogeneity drives diversity patterns (Montané, Casals & Dale, 2011). In this desert steppe community study, dominant fine scale effects exist, because of finer-scale processes of community assembly probably led to observed patterns of spatial autocorrelation in community responses (He et al., 2006). These finer-scale processes may include dispersal, competition, predation, redistribution of soil moisture and nutrients (Luzuriaga et al., 2012; Ren et al., 2015). Also, the intergrowth relationship among different species is significant in the desert plant community (Wang & Hu, 2001). Many plant species grow together to avoid disturbance or for favorable environmental conditions (He et al., 2006). In our study, perennials (e.g., *S. breviflora*) are considered to be nurse plants, which provide facilitative interactions in arid ecosystems because they can improve microenvironmental conditions for other annual plants (Quevedorobledo, Pucheta & Ribasfernández, 2010). These fine-scale processes may be the reasons for spatial autocorrelation displayed at fine scales. The intermediate disturbance hypothesis posits that species diversity is highest when intermediate levels of disturbance allow species coexistence by preventing dominance of one or few strong competitors (Connell, 1978). Meanwhile, high grazing intensity can also enhance species richness by suppressing dominant species, whereas low grazing intensity can increase species’ abundances (Klink et al., 2016). Grazing disturbance might explain why species richness showed greater spatial variation in grazed areas outside the fence than ungrazed areas inside the fence, whereas the opposite was observed for abundance (Fig. 2).

### Spatial patterns and controlling processes

Variation partitioning in plant species diversity helps us understand the ecological processes that contributed to the observed spatial variation in the community structure of *S. breviflora* desert steppe vegetation. Variation partitioning of species richness, Shanon diversity and abundance inside and outside the fence shared several common features (Table 4), which indicated similar underlying processes controlling vegetation dynamics in the *S. breviflora* desert steppe community. In general, environmental variables explained the most variance in community responses, pure spatial components playing a much smaller role. Although there was a large amount of unexplained variation, relatively high variance explained by environmental variables suggest that species diversity is influenced by the soil, aridity, and interference of shift sand in the desert region (Ali, Dicknson & Murphy, 2000). Among all environmental factors, soil water is a key factor determining species distribution at smaller scales in the desert region (He et al., 2006).

Furthermore, soil particle size is correlated with soil water retention. For example, soils with large particles and pores can hold large amounts of water but have poor retention. In contrast, clay and silt soils have low porosity and high water-retention (Wu et al., 2014). Therefore, soil particle-size distribution may play an important role in determining the soil water availability, which largely determines the structure of community assembly in arid and semi-arid habitats (Chesson et al., 2004; Montané, Casals & Dale, 2011). Indeed, we found that soil particle-size distribution explained a significant fraction of the total variance of plant species diversity (Tables S3, S4). Low spatial explanation may be due to chance and dispersal limitation processes, which regulate species arrival, which are then subject to environmental filtering at a particular site (Hillerislambers et al., 2012). There exist some possible explanations for the high unexplained components [d]. One is that a large amount of variability in *S. breviflora* desert steppe community could not be captured by environmental and spatial variables at finer-scales studies. Another possible explanation is sampling error, but this is unlikely because we surveyed exhaustively.

Finally, species specificity, niche differentiation and other processes controlling the community structure and composition of the *S. breviflora* desert steppe community could be invoked in explaining community assembly patterns. Environmental heterogeneity at a relatively small scale is a key factor which positively affects species diversity as well as influences species assembly in semi-arid environment (Bergholz et al., 2017). Grazing introduces more fine-scale environmental heterogeneity into grasslands, which may be beneficial for structural complexity and diversity (Jerrentrup et al., 2015). Heterogeneous environmental conditions may allow different species to occupy different microhabitats and provide ecological niches promoting diversity (Mayfield et al., 2010). This was confirmed as the spatially structured environmental variation contributed more to the three diversity variables in grazing areas (Table 4).

Restoration of degraded grassland in semi-arid region of China aims to improve production, strengthen ecological functioning, and promote historical conditions and traditional values. The ultimate goal of ecological restoration should be to maintain biological diversity. Species diversity is the commonly used parameter to assess restoration success (Waldén & Lindborg, 2016). For the present studied community, grazing exclusion may be an efficient way to restore grassland but this practice may sacrifice biodiversity. (Tables S1, S2). In order to obtain more precise estimates of restoration success, a clear restoration goal is essential at different stages of the restoration process. This will result in more efficient measures and ecological engineering to fulfill a specific aim of restoration.

## Conclusions

In a *S. breviflora* desert steppe community, relationships of species–area, abundance–area, and Shanon diversity–area were described as different mathematical functions. Species–area curves as the first pattern at the community level was better fitted by the logistic model rather than the power function. Excluding grazers for six years had significant effects on species diversity patterns at smaller scales. Similar underlying controlling processes on plant species diversity were observed between grazing and enclosure community. Plant species diversity spatial pattern of the *S. breviflora* desert steppe community may be predictable because of environmental factor has a large contribution on species diversity. Among all environmental factors, soil particle size distribution was closely relation to plant species diversity. Furthermore, soil particle size distribution may play an important role in determining the soil water availability. Thus, for the present studied community, further research should examined the relationships between species diversity and soil water availability.

##  Supplemental Information

10.7717/peerj.4359/supp-1 Data S1 Dataset

10.7717/peerj.4359/supp-2 Figure S1 Species-area curves inside and outside the fence Two saturation points at plot size 0.5 m^2^ and 5 m^2^ were determined, respectively. Three nested quadrat area were established in grazing and fenced treatments. Each nested quadrat area was divided into 13 (0.01, 0.02, 0.04, 0.08, 0.16, 0.32, 0.64, 164 1.28, 2.56, 5.12, 10.24, 20.48, 40.96 m^2^) subplots for measurements of species-area curves. Species presence and absence were recorded (ranging in size from 0.01 to 40.96 m^2^).

10.7717/peerj.4359/supp-3 Figure S2 Power, Exponential and Logical models fitted to the species richness-area relations outside and inside the fence Species richness in the SAR curves increased with sampling area and slopes decreased with increasing sampling area both inside and outside the fence. Both the sampling areas, 5 m^2^ (inside) and 2.5 m^2^ (outside), had approximately 20 species.

10.7717/peerj.4359/supp-4 Figure S3 Abundance and density-area curves outside and inside the fence The abundance-area curves were relatively similar inside and outside the fence and the curves were well fitted by the linear function. The density-area relationship showed that the density observed at smaller spatial scales varied greatly inside and outside the fence.

10.7717/peerj.4359/supp-5 Figure S4 Power, Exponential, Logical models and parabolic model fitted to the Shannon diversity-area relations outside and inside the fence The relation between area and Shannon-diversity varied little inside and outside the fence for all sampling scales.

10.7717/peerj.4359/supp-6 Table S1 Changes in species diversity, biomass between the two sites (inside, outside). Plant diversity of grassland was assessed using the Richness index (*R*), Shannon-Wiener index (*H*′), Simpson index (*D*), and Pielou index (*J*). Biomass Measurements of plant diversity and biomass showed significant (*P* < 0.05) difference between the two sites (inside, outside). The plant diversity was lower inside the exclusion. The biomass decreased for *Leguminosae*, *Compositae* and *weeds* and increased for grass and total biomass inside the fence.

10.7717/peerj.4359/supp-7 Table S2 Soil organic carbon content and particle size distribution to a depth of 40cm inside and outside the fence. Differences in mean diversity between two sites were assessed with paired-sample *T*-tests The difference between soil organic carbon (SOC) contents wasn’t statistically significant in the inside versus the outside of the fence. SOC increased at depths of 0–5 and 5–10 cm, and declined at depths of 10–20 and 20–40 cm when compared with the outside of the fence. The soil particle contents inside the fence, such as clay (<0.002 mm) content, was significantly (*p* < 0.05) increased at depth of 0–20 cm but decreased at a depth of 20–40 cm. The silt content at depths of 0–5, 5–10 and 20–40 cm exhibited differences similar to those of the clay contents inside the fence. The fine sand (0.1–0.05 mm) content decreased significantly (*p* < 0.05) at depths of 0–5, 5–10 cm but increased significantly (*p* < 0.05) at depths of 20–40 cm compared to the outside of the fence. Inside the fence had higher coarse sand content (2–0.1 mm) at all depths than the outside, but only at depth of 20–40 cm was statistically significant (*p* < 0.05).

10.7717/peerj.4359/supp-8 Table S3 Explanatory environmental and spatial variables selected by the forward selective procedure in the redundancy analyses outside the fence PCNM, Pricncipal coordinates of neighbor matrices. AdjR^2^ Cum refers to adjusted cumulative square of the sum of all canonical eigenvalues (expressing explaind variance). *F* refers to *F* test statistic. *P* value refers to the significance of the variable (Monte Carlo permutation on test). We used forward selection for all environmental and PCNM variables, to obtain the significant environmental and PCNM variables, respectively. Outside the fence, the silt was the main environmental variables.

10.7717/peerj.4359/supp-9 Table S4 Explanatory environmental and spatial variables selected by the forward selective procedure in the redundancy analyses inside the fence PCNM=Pricncipal coordinates of neighbor matrices. AdjR^2^ Cum refers to adjusted cumulative square of the sum of all canonical eigenvalues (expressing explaind variance). *F* refers to *F* test statistic. *P* value refers to the significance of the variable (Monte Carlo permutation on test). We used forward selection for all environmental and PCNM variables, to obtain the significant environmental and PCNM variables, respectively. Inside the exclusion, the coarse sand was the major environmental factors.

## Additional Information and Declarations

### Data Availability

The following information was supplied regarding data availability: The raw data is provided in a Supplemental File.
