# Supplementary figures and images for: A six-year grazing exclusion changed plant species diversity of a Stipa breviflora desert steppe community, northern China
Source: PeerJ. 2018 Feb 13;6:e4359. doi: 10.7717/peerj.4359 (PMC5815336; doi:10.7717/peerj.4359)

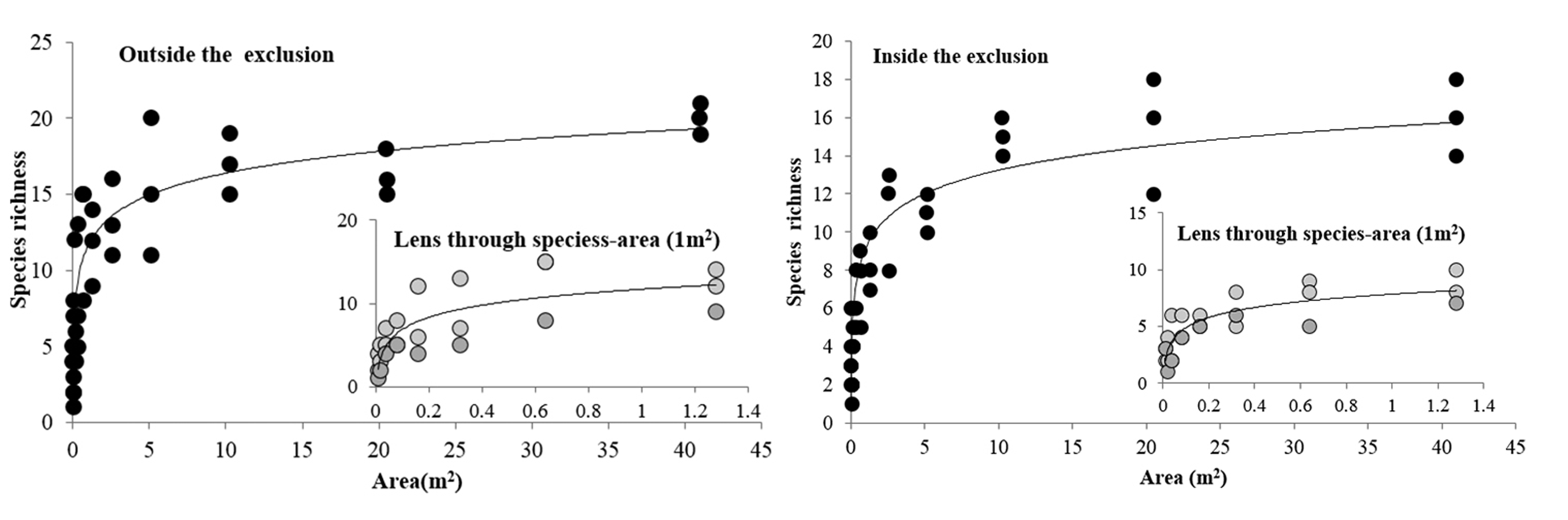

Supplement: Figure S1 — Two saturation points at plot size 0.5 m2 and 5 m2 were determined, respectively. Three nested quadrat area were established in grazing and fenced treatments. Each nested quadrat area was divided into 13 (0.01, 0.02, 0.04, 0.08, 0.16, 0.32, 0.64, 164 1.28, 2.56, 5.12, 10.24, 20.48, 40.96 m2) subplots for measurements of species-area curves. Species presence and absence were recorded (ranging in size from 0.01 to 40.96 m2). [file peerj-06-4359-s002.png]

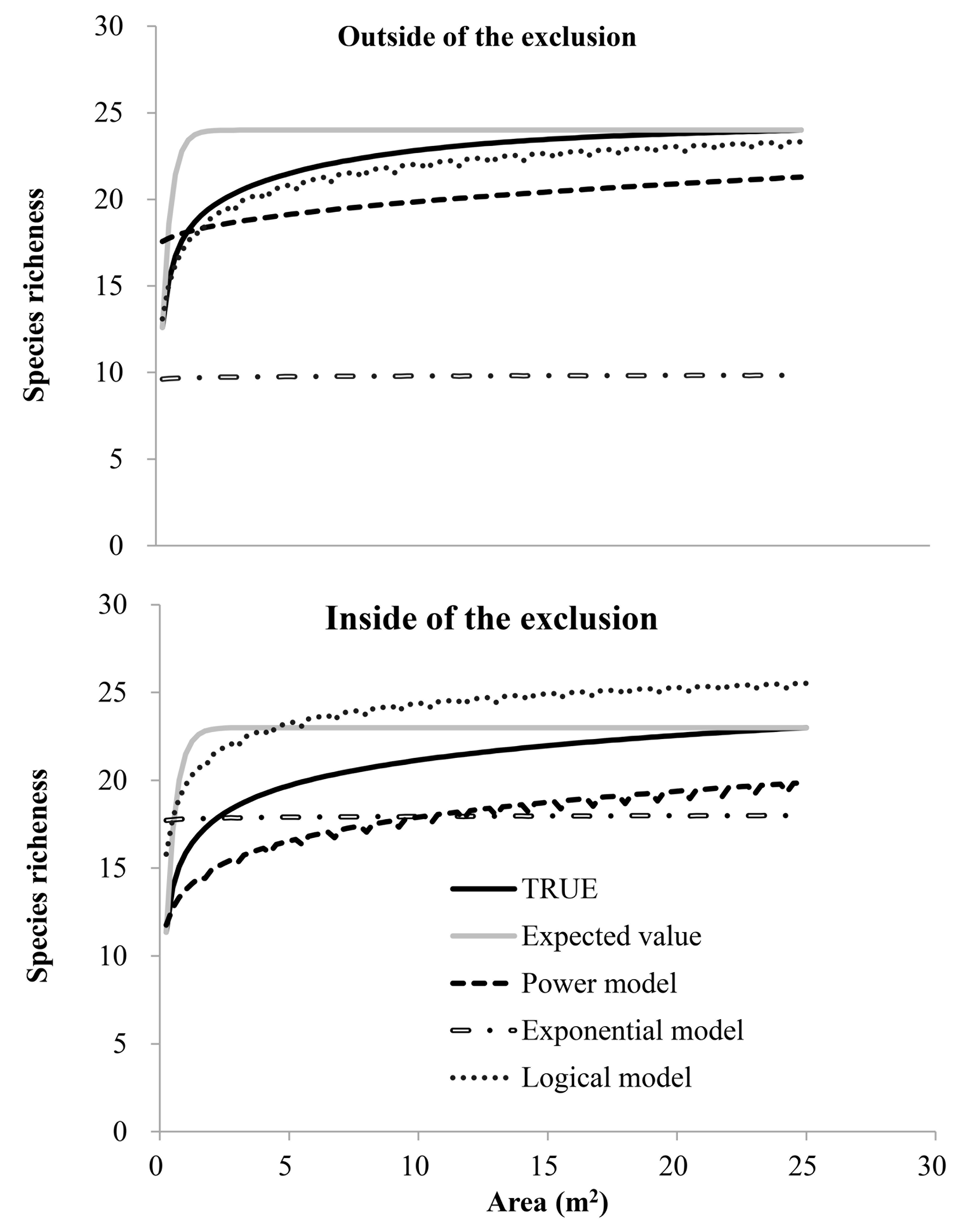

Supplement: Figure S2 — Species richness in the SAR curves increased with sampling area and slopes decreased with increasing sampling area both inside and outside the fence. Both the sampling areas, 5 m2 (inside) and 2.5 m2 (outside), had approximately 20 species. [file peerj-06-4359-s003.png]

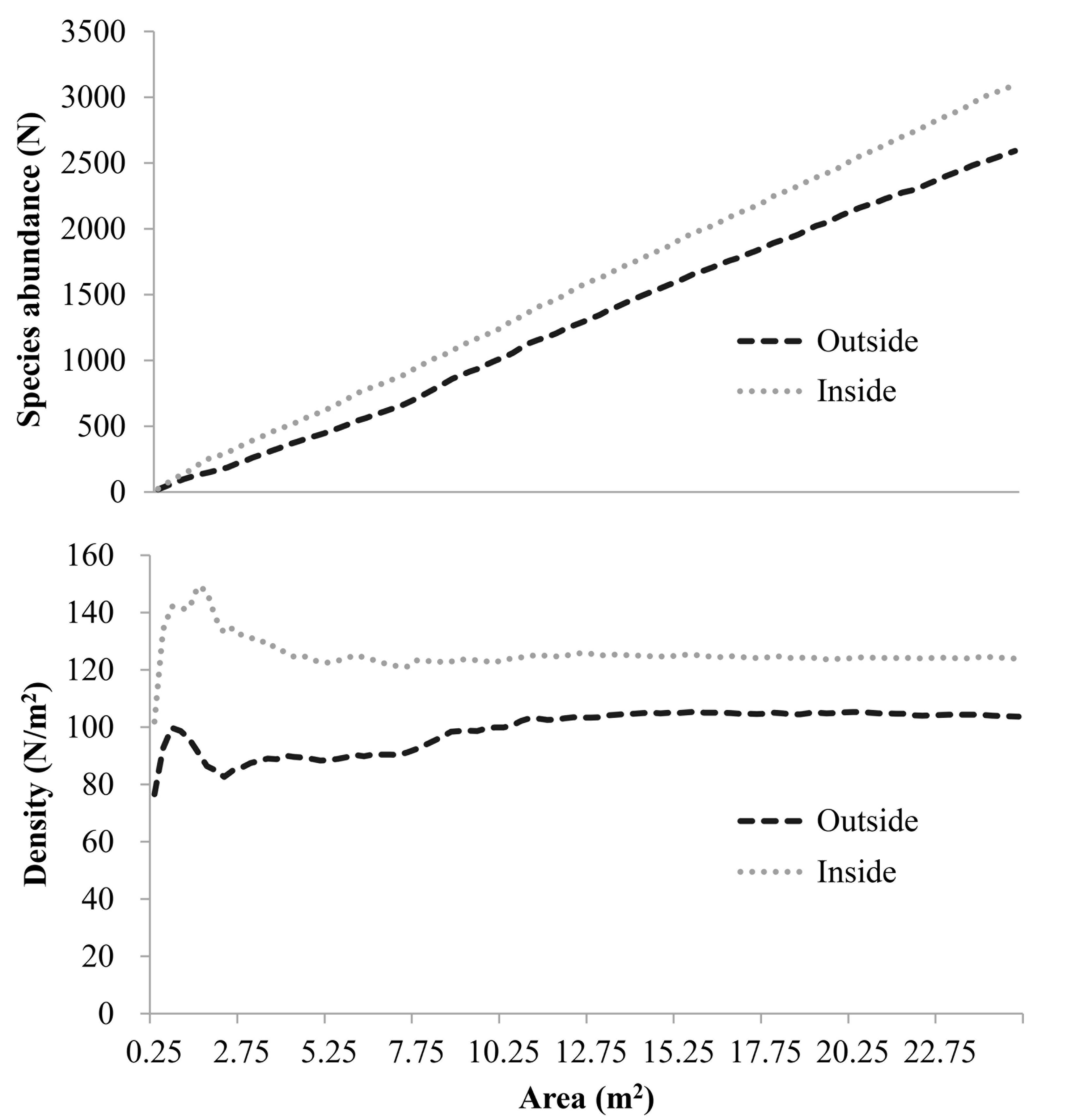

Supplement: Figure S3 — The abundance-area curves were relatively similar inside and outside the fence and the curves were well fitted by the linear function. The density-area relationship showed that the density observed at smaller spatial scales varied greatly inside and outside the fence. [file peerj-06-4359-s004.png]

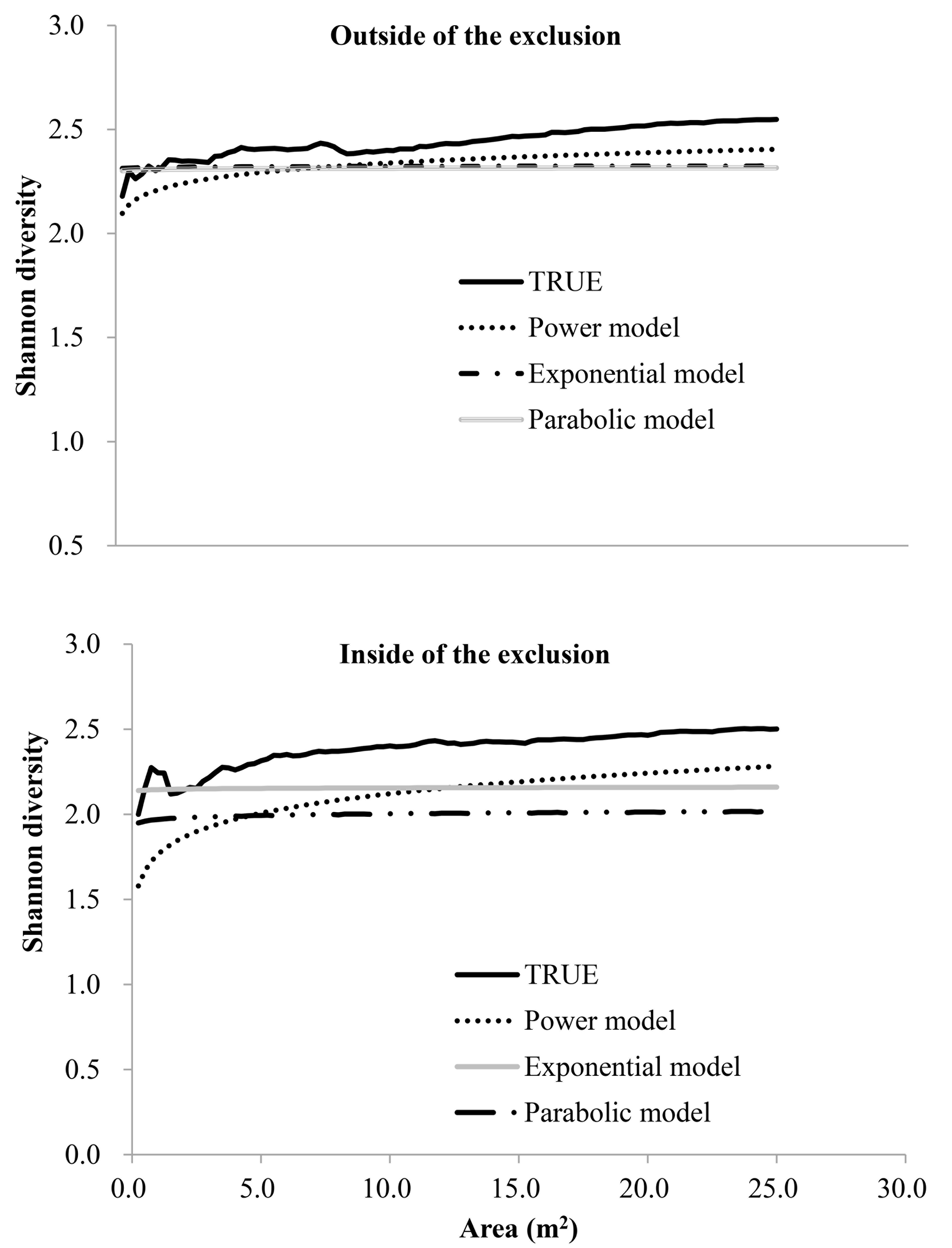

Supplement: Figure S4 — The relation between area and Shannon-diversity varied little inside and outside the fence for all sampling scales. [file peerj-06-4359-s005.png]
