# Supplementary material for: A six-year grazing exclusion changed plant species diversity of a Stipa breviflora desert steppe community, northern China
Source: PeerJ. 2018 Feb 13;6:e4359. doi: 10.7717/peerj.4359 (PMC5815336; doi:10.7717/peerj.4359)
Supplement: Table S1 — Measurements of plant diversity and biomass showed significant (P < 0.05) difference between the two sites (inside, outside). The plant diversity was lower inside the exclusion. The biomass decreased for Leguminosae, Compositae and weeds and increased for grass and total biomass inside the fence. [file peerj-06-4359-s006.docx]

**Preliminary sampling** **for vegetation and soil inside and outside the fence**

Five (1 m×1 m) randomly distributed subplots were established in each 50 × 50 m plot in grazing and fenced treatments. The above-ground part of the green plants were cut, and five plant indices comprising the number of species, plant height, cover and abundance for each plant species were recorded. At the same time, three random soil samples in each quadrat were collected with a 5-cm diameter soil auger from four depths: 0-5 cm, 5-10 cm, 10-20 cm and 20-40 cm. Soil samples at the same depth in each subplot were then mixed to a single sample. The results showed in Table S1 and Table S2

**Table S1** Changes in species diversity, biomass between the two sites (inside, outside). Plant diversity of grassland was assessed using the Richness index (*R*), Shannon-Wiener index (*H’*), Simpson index (*D*), and Pielou index (*J*). Biomass as measured by grass, *Leguminosae*, *Compositae* and *weeds* for measurements. Diﬀerences in mean diversity between two sites were assessed with paired-sample T-tests.

| **Sites** | **Diversity index** | | | | **Biomass of different family** | | | | |
| --- | --- | --- | --- | --- | --- | --- | --- | --- | --- |
|  | ***R*** | ***H'*** | ***D*** | ***J*** | ***Grass*** | ***Leguminosae*** | ***Compositae*** | ***Ruderal*** | **total biomass** |
| **Outside** | 13.73±1.87 | 10.14±1.21 | 8.04±0.94 | 0.88±0.03 | 43.57±10.23 | 37.84±16.75 | 5.24±5.62 | 37.79±11.86 | 124.44±28.49 |
| **Inside** | 10.73±1.58 | 7.41±1.28 | 5.5±1.22 | 0.84±0.04 | 74.14±18.99 | 11.89±6.24 | 1.11±1.21 | 18.99±11.98 | 106.13±18.69 |
| ***t test*** | *p*=0.00 | *p* =0.00 | *p* =0.00 | *p* =0.00 | *p* =0.00 | *p* =0.00 | *p* =0.03 | *p* =0.00 | *p* =0.05 |

Measurements of plant diversity and biomass showed significant (P<0.05) difference between the two sites (inside, outside). The plant diversity was lower inside the exclusion. The biomass decreased for *Leguminosae*, *Compositae* and *weeds* and increased for grass and total biomass inside the fenced exclosure.
