# Supplementary material for: A six-year grazing exclusion changed plant species diversity of a Stipa breviflora desert steppe community, northern China
Source: PeerJ. 2018 Feb 13;6:e4359. doi: 10.7717/peerj.4359 (PMC5815336; doi:10.7717/peerj.4359)
Supplement: Table S2 — The difference between soil organic carbon (SOC) contents wasn’t statistically significant in the inside versus the outside of the fence. SOC increased at depths of 0–5 and 5–10 cm, and declined at depths of 10–20 and 20–40 cm when compared with the outside of the fence. The soil particle contents inside the fence, such as clay (<0.002 mm) content, was significantly (p < 0.05) increased at depth of 0–20 cm but decreased at a depth of 20–40 cm. The silt content at depths of 0–5, 5–10 and 20–40 cm exhibited differences similar to those of the clay contents inside the fence. The fine sand (0.1–0.05 mm) content decreased significantly (p < 0.05) at depths of 0–5, 5–10 cm but increased significantly (p < 0.05) at depths of 20–40 cm compared to the outside of the fence. Inside the fence had higher coarse sand content (2–0.1 mm) at all depths than the outside, but only at depth of 20–40 cm was statistically significant (p < 0.05). [file peerj-06-4359-s007.docx]

**Preliminary sampling** **for vegetation and soil inside and outside the fence**

Five (1 m×1 m) randomly distributed subplots were established in each 50 × 50 m plot in grazing and fenced treatments. The above-ground part of the green plants were cut, and five plant indices comprising the number of species, plant height, cover and abundance for each plant species were recorded. At the same time, three random soil samples in each quadrat were collected with a 5-cm diameter soil auger from four depths: 0-5 cm, 5-10 cm, 10-20 cm and 20-40 cm. Soil samples at the same depth in each subplot were then mixed to a single sample. The results showed in Table S1 and Table S2

**Table S2** Soil organic carbon content and particle size distribution to a depth of 40cm inside and outside the fence. Diﬀerences in mean diversity between two sites were assessed with paired-sample T-tests.

| Parameter | Sites | Soil depth/cm | | | |
| --- | --- | --- | --- | --- | --- |
|  |  | 0-5cm | 5-10cm | 10-20cm | 20-40cm |
| Soil organic carbon (%) | Outside | 0.27±0.07 | 0.27±0.09 | 0.4±0.12 | 0.32±0.12 |
|  | Inside | 0.29±0.07 | 0.34±0.22 | 0.37±0.11 | 0.27±0.08 |
|  | *t test(p value)* | 0.41 | 0.27 | 0.58 | 0.12 |
| Clay (<0.002mm) (%) | Outside | 2.06±0.48 | 2.92±0.73 | 4.96±1.35 | 5.36±0.92 |
|  | Inside | 2.44±0.69 | 4.36±1.85 | 7.23±1.78 | 2.34±0.96 |
|  | *t test(p value)* | 0.10 | 0.01 | 0.00 | 0.00 |
| Silt (0.05-0.002mm) (%) | Outside | 18.52±3.63 | 25.37±6.75 | 41.41±9.82 | 44.22±8.09 |
|  | Inside | 19.79±4.88 | 30.95±7.99 | 44.31±4.09 | 19.83±7.4 |
|  | *t test(p value)* | 0.46 | 0.06 | 0.31 | 0.00 |
| Fine sand (0.1-0.05mm) (%) | Outside | 35.35±1.21 | 31.75±2.33 | 24.94±4.11 | 23.7±3.47 |
|  | Inside | 32.43±3.2 | 28.16±3.74 | 22.94±2.18 | 34.5±3.63 |
|  | *t test(p value)* | 0.01 | 0.00 | 0.14 | 0.00 |
| Coarse sand (2-0.1mm) (%) | Outside | 44.06±3.5 | 39.97±5.33 | 28.69±6.96 | 26.72±5.72 |
|  | Inside | 45.34±4.45 | 36.53±6.03 | 25.51±3.33 | 43.33±5.77 |
|  | *t test(p value)* | 0.38 | 0.15 | 0.12 | 0.00 |

The difference between soil organic carbon (SOC) contents wasn’t statistically significant in the inside versus the outside of the fence. SOC increased at depths of 0-5 and 5-10 cm, and declined at depths of 10-20 and 20-40 cm when compared with the outside of the fence. The soil particle contents inside the fence, such as clay (<0.002 mm) content, was significantly (*p*<0.05) increased at depth of 0-20 cm but decreased at a depth of 20-40 cm. The silt content at depths of 0-5, 5-10 and 20-40 cm exhibited differences similar to those of the clay contents inside the fence. The fine sand (0.1-0.05 mm) content decreased significantly (*p*<0.05) at depths of 0-5, 5-10 cm but increased significantly (*p*<0.05) at depths of 20-40 cm compared to the outside of the fence. Inside the fence had higher coarse sand content (2-0.1 mm) at all depths than the outside, but only at depth of 20-40 cm was statistically significant (*p*<0.05).
