# Supplementary material for: A six-year grazing exclusion changed plant species diversity of a Stipa breviflora desert steppe community, northern China
Source: PeerJ. 2018 Feb 13;6:e4359. doi: 10.7717/peerj.4359 (PMC5815336; doi:10.7717/peerj.4359)
Supplement: Table S3 — PCNM, Pricncipal coordinates of neighbor matrices. AdjR2 Cum refers to adjusted cumulative square of the sum of all canonical eigenvalues (expressing explaind variance). F refers to F test statistic. P value refers to the significance of the variable (Monte Carlo permutation on test). We used forward selection for all environmental and PCNM variables, to obtain the significant environmental and PCNM variables, respectively. Outside the fence, the silt was the main environmental variables. [file peerj-06-4359-s008.docx]

**Table S3** Explanatory environmental and spatial variables selected by the forward selective procedure in the redundancy analyses outside the fence

|  | **variables** | **Order** | **Variables** | **R^2^** | ***R^2^Cum*** | ***Adj R^2^Cum*** | ***F*** | ***pval*** |
| --- | --- | --- | --- | --- | --- | --- | --- | --- |
| **Shannon diversity** | **Environment** | 1 | Silt | 0.88 | 0.88 | 0.88 | 741.74 | 0.0001 |
|  |  | 2 | Coarse sand | 0.05 | 0.93 | 0.93 | 74.19 | 0.0001 |
|  |  | 3 | Fine sand | 0.01 | 0.94 | 0.94 | 8.83 | 0.0031 |
|  | **Space** | 1 | PCNM4 | 0.15 | 0.15 | 0.14 | 17.32 | 0.0010 |
|  |  | 2 | PCNM20 | 0.08 | 0.23 | 0.21 | 9.82 | 0.0050 |
|  |  | 3 | PCNM9 | 0.06 | 0.29 | 0.27 | 8.44 | 0.0040 |
|  |  | 4 | PCNM8 | 0.06 | 0.35 | 0.32 | 8.13 | 0.0060 |
|  |  | 5 | PCNM2 | 0.04 | 0.39 | 0.36 | 6.72 | 0.0100 |
|  |  | 6 | PCNM21 | 0.04 | 0.43 | 0.39 | 6.39 | 0.0170 |
|  |  | 7 | PCNM10 | 0.04 | 0.47 | 0.43 | 6.53 | 0.0160 |
|  |  | 8 | PCNM17 | 0.03 | 0.50 | 0.45 | 5.56 | 0.0220 |
|  |  | 9 | PCNM40 | 0.03 | 0.52 | 0.48 | 4.83 | 0.0330 |
|  |  | 10 | PCNM14 | 0.03 | 0.55 | 0.50 | 4.96 | 0.0310 |
| **Species richness** | **Environment** | 1 | Silt | 0.79 | 0.79 | 0.79 | 363.41 | 0.0001 |
|  |  | 2 | Coarse sand | 0.04 | 0.83 | 0.83 | 23.65 | 0.0001 |
|  | **Space** | 1 | PCNM4 | 0.14 | 0.14 | 0.13 | 15.30 | 0.0010 |
|  |  | 2 | PCNM8 | 0.07 | 0.20 | 0.19 | 8.42 | 0.0080 |
|  |  | 3 | PCNM20 | 0.06 | 0.26 | 0.24 | 7.31 | 0.0060 |
|  |  | 4 | PCNM9 | 0.06 | 0.32 | 0.29 | 7.71 | 0.0100 |
|  |  | 5 | PCNM2 | 0.04 | 0.35 | 0.32 | 5.60 | 0.0240 |
|  |  | 6 | PCNM28 | 0.04 | 0.39 | 0.35 | 5.46 | 0.0210 |
|  |  | 7 | PCNM21 | 0.03 | 0.42 | 0.38 | 5.26 | 0.0270 |
|  |  | 8 | PCNM17 | 0.03 | 0.46 | 0.41 | 5.37 | 0.0150 |
|  |  | 9 | PCNM10 | 0.03 | 0.49 | 0.43 | 5.37 | 0.0220 |
|  |  | 10 | PCNM40 | 0.03 | 0.52 | 0.46 | 5.51 | 0.0250 |
|  |  | 11 | PCNM14 | 0.03 | 0.54 | 0.48 | 4.89 | 0.0300 |
| **Abundance** | **Environment** | 1 | Silt | 0.57 | 0.57 | 0.57 | 132.31 | 0.0001 |
|  |  | 2 | Coarse sand | 0.04 | 0.62 | 0.61 | 10.43 | 0.0014 |
|  |  | 3 | Cover | 0.05 | 0.66 | 0.65 | 13.97 | 0.0008 |
|  |  | 4 | Density | 0.05 | 0.71 | 0.70 | 16.01 | 0.0002 |
|  | **Space** | 1 | PCNM9 | 0.13 | 0.13 | 0.12 | 14.72 | 0.0010 |
|  |  | 2 | PCNM21 | 0.07 | 0.20 | 0.19 | 8.99 | 0.0040 |
|  |  | 3 | PCNM14 | 0.05 | 0.26 | 0.24 | 7.01 | 0.0150 |
|  |  | 4 | PCNM2 | 0.05 | 0.31 | 0.28 | 7.13 | 0.0110 |
|  |  | 5 | PCNM20 | 0.05 | 0.36 | 0.33 | 7.25 | 0.0090 |
|  |  | 6 | PCNM18 | 0.05 | 0.41 | 0.37 | 7.50 | 0.0070 |
|  |  | 7 | PCNM8 | 0.04 | 0.45 | 0.41 | 7.45 | 0.0110 |

PCNM=Pricncipal coordinates of neighbor matrices. AdjR^2^Cum refers to adjusted cumulative square of the sum of all canonical eigenvalues (expressing explaind variance). F refers to F test statistic. P value refers to the significance of the variable (Monte Carlo permutation on test ). We used forward selection for all environmental and PCNM variables, to obtain the significant environmental and PCNM variables, respectively. Outside the fence, the silt was the main environmental variables.
