# Supplementary material for: A six-year grazing exclusion changed plant species diversity of a Stipa breviflora desert steppe community, northern China
Source: PeerJ. 2018 Feb 13;6:e4359. doi: 10.7717/peerj.4359 (PMC5815336; doi:10.7717/peerj.4359)
Supplement: Table S4 — PCNM=Pricncipal coordinates of neighbor matrices. AdjR2 Cum refers to adjusted cumulative square of the sum of all canonical eigenvalues (expressing explaind variance). F refers to F test statistic. P value refers to the significance of the variable (Monte Carlo permutation on test). We used forward selection for all environmental and PCNM variables, to obtain the significant environmental and PCNM variables, respectively. Inside the exclusion, the coarse sand was the major environmental factors. [file peerj-06-4359-s009.docx]

**Table S4** Explanatory environmental and spatial variables selected by the forward selective procedure in the redundancy analyses inside the fence

| **Inside exclusion** | **Variables** | **Order** | **Variables** | **R^2^** | ***R^2^Cum*** | ***Adj R^2^Cum*** | ***F*** | ***pval*** |
| --- | --- | --- | --- | --- | --- | --- | --- | --- |
| **Shannon diversity** | **Environment** | 1 | Coarse sand | 0.72 | 0.72 | 0.72 | 253.60 | 0.0001 |
|  |  | 2 | Silt | 0.17 | 0.89 | 0.89 | 145.79 | 0.0001 |
|  |  | 3 | Fine sand | 0.01 | 0.90 | 0.89 | 7.62 | 0.0072 |
|  |  | 4 | Cover | 0.01 | 0.90 | 0.90 | 7.08 | 0.0091 |
|  | **Space** | 1 | PCNM25 | 0.08 | 0.08 | 0.07 | 8.09 | 0.001 |
|  |  | 2 | PCNM35 | 0.06 | 0.14 | 0.12 | 7.31 | 0.007 |
|  |  | 3 | PCNM13 | 0.05 | 0.19 | 0.16 | 5.81 | 0.021 |
|  |  | 4 | PCNM6 | 0.05 | 0.24 | 0.21 | 6.10 | 0.015 |
|  |  | 5 | PCNM39 | 0.04 | 0.28 | 0.24 | 5.43 | 0.023 |
| **Speciess diversity** | **Environment** | 1 | Coarse sand | 0.60 | 0.60 | 0.59 | 145.77 | 0.0001 |
|  |  | 2 | Silt | 0.16 | 0.76 | 0.75 | 62.23 | 0.0001 |
|  | **Space** | 1 | PCNM35 | 0.07 | 0.07 | 0.06 | 7.25 | 0.011 |
|  |  | 2 | PCNM39 | 0.07 | 0.14 | 0.12 | 7.52 | 0.004 |
|  |  | 3 | PCNM25 | 0.05 | 0.19 | 0.16 | 6.30 | 0.011 |
|  |  | 4 | PCNM13 | 0.04 | 0.23 | 0.20 | 5.24 | 0.03 |
|  |  | 5 | PCNM28 | 0.04 | 0.27 | 0.23 | 5.25 | 0.024 |
| **Abundance** | **Environment** | 1 | Coarse sand | 0.47 | 0.47 | 0.47 | 87.62 | 0.0001 |
|  |  | 2 | Silt | 0.11 | 0.58 | 0.57 | 25.72 | 0.0001 |
|  | **Space** | 1 | PCNM28 | 0.07 | 0.07 | 0.06 | 7.29 | 0.013 |
|  |  | 2 | PCNM39 | 0.06 | 0.13 | 0.11 | 6.85 | 0.012 |

PCNM=Pricncipal coordinates of neighbor matrices. AdjR^2^Cum refers to adjusted cumulative square of the sum of all canonical eigenvalues (expressing explaind variance). F refers to F test statistic. P value refers to the significance of the variable (Monte Carlo permutation on test )

**Forward selection for environmental and spatial factors.** We used forward selection for all environmental and PCNM variables, to obtain the significant environmental and PCNM variables, respectively. Inside the exclusion, the coarse sand was the major environmental factors extracted (Table S3, Table S4).
